# Factors Associated with Compassion Fatigue in Assistance Animal Trainers in Australia—A Qualitative Investigation

**DOI:** 10.3390/ani15030337

**Published:** 2025-01-24

**Authors:** James Verrall, Vanessa Rohlf, Tiffani J. Howell

**Affiliations:** 1 School of Psychology and Public Health, La Trobe University, Bundoora, VIC 3086, Australia; 20261990@students.latrobe.edu.au (J.V.); v.rohlf@latrobe.edu.au (V.R.); 2 School of Psychology and Public Health, La Trobe University, Bendigo, VIC 3552, Australia

**Keywords:** service dog, secondary traumatic stress, guide dog, burnout, professional quality of life, workplace wellbeing

## Abstract

Assistance animal (AA) trainers could develop compassion satisfaction (CS) but also compassion fatigue (CF), due to potential exposure to suffering through working with people with disabilities. The negative outcomes associated with CF could result in fewer trainers and longer wait times for people in need. Using the Job Demands–Resources Model (JD-R), this study aimed to qualitatively identify the demands and resources of AA trainers and explore signs of CF and CS. Six trainers participated in semi-structured interviews, and five themes were identified. The Demands theme represented challenges trainers experienced, such as concerns about animal welfare and client wellbeing. The Resources theme represented personal characteristics and job aspects that assisted the trainers, like resilience and formal support from the employer. Negative Impacts included signs of CF and represented how participants were negatively affected by their job. Similarly, Positive Impacts included signs of CS and represented how participants were positively affected. Misguided Trainer Expectations represented the expectations trainers had starting the job compared to the reality of the role. The discovery of CF signs provides evidence of a previously unidentified psychosocial hazard for trainers. These findings should be used by training organizations to better support trainers.

## Simple Summary

Compassion fatigue refers to the negative psychological toll associated with caring for others. It is a common issue for people working in the helping professions, such as nurses and aged care workers. It has also been observed in people working with animals, like veterinarians. Assistance animal (AA) trainers may also be at risk of compassion fatigue, because they work closely with both animals and vulnerable people, training animals to support people with disabilities. However, research into AA trainers is limited. We interviewed six trainers to identify the stressors (i.e., demands) inherent in their job, but also what helps them do their job well (i.e., resources). Demands included concerns about animal welfare, client wellbeing, and navigating an industry with very little regulation. Resources included personal characteristics like resilience, but also support from their employer, like mental health first aid training. We also found signs of compassion fatigue and its counterpoint, compassion satisfaction, where people experience positive feelings about their work. Future research should adapt these findings into a large-scale, quantitative study with similar aims. Assistance animal training organizations can use this knowledge to implement programs aimed at enhancing wellbeing and reducing stress for their trainers.

## 1. Introduction

Assistance animals (AAs) are trained animals that help their handler (i.e., the person requiring assistance) to manage a range of disability types [1]. This includes hearing and vision impairments [2], mobility impairments [3], developmental disorders [4], and psychiatric disabilities such as post-traumatic stress disorder (PTSD) [5]. AA trainers train both the animal and handler in a process that may take several years [6]. For the animal, this will include obedience training and disability-specific task training; for the handler, this will include how to work with the AA to mitigate the impact of their disability, and how to meet the animal’s welfare needs. Due to their role in educating and training individuals, AA trainers are considered helping professionals [7]. They work very closely with their clients, often over a long period of time, and sometimes in the client’s own community and home, so it stands to reason that they would learn a lot about that person’s life and history.

Prior research investigating the experiences of helping professionals has identified positive and negative aspects of helping. The positive aspects can lead to the development of compassion satisfaction (CS); however, the negative aspects can result in the development of compassion fatigue (CF) [8]. CF risk is particularly high when working with individuals who are suffering or have experienced trauma [9]. AA trainers may help both individuals who are perceived to be suffering [10] and who have experienced trauma [5]. Therefore, there is potential for AA trainers to experience CS but there is also a risk that they may develop CF. Despite this, there has been very little research exploring the experiences of AA trainers and how they are impacted by their work.

CS includes feelings such as pleasure or fulfillment which can be derived from feeling positively about colleagues, the ability to contribute to an individual in need, or one’s contribution to the workplace and wider community [8]. It has been observed in other helping professionals, such as nurses [11], therapists [12], and veterinarians [13]. CS can have a protective effect against CF [12] and can result in better work performance, better employee engagement, and a more positive work environment [14].

On the other hand, CF is a state of exhaustion and preoccupation with the suffering of others [8]. CF was originally defined by Figley [15] as secondary traumatic stress (STS), which is a stress response resulting in symptoms such as invasive thoughts, nightmares, avoidance, and hypervigilance [15]. It is experienced from witnessing or hearing of another person’s trauma or suffering [15].

Work by Stamm [8] has built upon the definition of CF to include symptoms of burnout. Burnout is a progressive exhaustion due to prolonged stressors in the workplace, resulting in cynicism and feelings of inefficacy [8]. Thus, according to Stamm [8], CF is the combined effect of both burnout and STS. CF has been observed in other helping professionals, such as nurses [16], care workers [17], and mental health practitioners [18], as well as animal care professionals such as veterinarians and animal care workers [19].

CF has been associated with suboptimal patient care [20], less job satisfaction [21], higher rates of staff turnover [16], and higher rates of mental health problems [22]. Given these negative individual and workplace outcomes, exploring whether this phenomenon is experienced by AA trainers is important so that prevention programs can be put in place to protect workers. Previous research investigating the experiences of AA trainers has identified five main themes affecting trainers [23]. These were discrimination against handlers, lack of regulation, lack of disability-specific education, difficulties in interprofessional collaboration, and funding barriers. These themes represented the main barriers trainers faced in attempting to complete their job. This study focused on service delivery factors rather than what trainers found challenging and rewarding regarding interactions with clients, and how they were psychologically impacted. Therefore, Hill et al. [23] did not capture any signs of CF and CS or any experiences which might lead to the development of these outcomes. This leaves a gap in the literature regarding the exploration of experiences that AA trainers find challenging and rewarding, how they are impacted by them, and whether there is a potential for CF and CS.

Addressing the knowledge gap can be achieved through an exploratory study; thus, qualitative research was indicated as the first step [24]. To assist in the identification of challenging and rewarding AA trainer experiences, a job stress model can be used to provide a theoretical framework [25]. One such framework that has been used in the past for exploring factors related to CF [9] is the Job Demands–Resource model (JD-R) [26]. The JD-R is one of the most widely used models for understanding job stress [25] and has demonstrated consistent validity across various occupational groups in different countries [26].

The JD-R categorizes all work-related factors as either job demands or resources [26]. Job demands are the aspects of a job that require physical, cognitive, or emotional effort and incur physiological or psychological costs [27]. Examples of job demands include high workload, time pressure, emotionally demanding situations, and cognitively straining tasks [26]. Resources, on the other hand, are personal factors or job aspects that motivate and energize workers, assisting them to better meet or cope with job demands [26]. Resources include personal resources, such as resilience, self-efficacy, and assertiveness [26], and job resources, such as opportunities for professional development, supervisor feedback, and job autonomy [27]. The JD-R model proposes that health problems arise in workers when perceived job demands are too high for a prolonged period and there are inadequate perceived resources to meet demands [27]. This can result in exhaustion, withdrawal behaviors, and other psychological difficulties [28].

The JD-R has been used to analyze and predict CF and CS based on the reported job demands and resources for mental health professionals [9]. In a systematic review that identified the demands associated with CF and the resources associated with CS, mental strain from working with clients who experience traumatic symptoms was a significant predictor of both burnout and STS, and support from supervisors and coworkers significantly predicted CS [9]. This study demonstrates how the JD-R can provide a theoretical framework by allowing for the categorization of AA trainers’ experiences as either demands or resources, while also permitting exploration for signs of CF and CS.

Given the potential for exposure to trauma and the previous literature indicating that helping professionals are at risk of developing CF, it is possible that CF exists among this population. AA trainers could be experiencing the negative outcomes associated with CF, resulting in fewer trainers due to attrition from the industry, and therefore longer wait times for people in need, as well as lower quality of life for trainers who remain in the industry. The aim of this qualitative study was to identify job demands and resources and explore whether signs of CF and CS exist in AA trainers.

## 2. Materials and Methods

This study received approval from La Trobe University Human Research Ethics Committee (approval number HEC24224; approved 23 July 2024).

### 2.1. Participants

A convenience sample of six participants took part in the study; four were female and two were male (Table 1). Recruitment was carried out through email distribution of advertising material to publicly available email addresses of AA training organizations. Interested individuals were also asked to pass on the advertising materials to their colleagues, resulting in a snowball method of recruitment. People were eligible to participate if they were at least 18 years of age and had worked in Australia as an AA trainer in the past 12 months. All participants worked exclusively with dogs. Two participants worked for the same large training organization and worked only with guide dogs, while the other four trainers worked independently or with smaller organizations, and trained a range of assistance dogs, including psychiatric assistance, mobility assistance, and medical alert dogs. Only one participant worked part-time with the rest either working full-time or self-employed. No other demographic data were collected from participants.

### 2.2. Materials

A semi-structured interview schedule was developed for this study, which consisted of 12 open-ended questions, with additional prompts if required. Questions explored participants’ work motivations, perceived challenges, stressful or fatiguing aspects of their work, and rewarding aspects and suggestions for workplace wellbeing interventions. To reduce participant priming, the terms CF and CS were not used on any advertising material or during the interview, until the final two questions, in which participants were given a brief definition of CF and CS and were asked for their thoughts about these conditions in relation to AA trainers. Questions were designed to allow participants the opportunity to discuss what they felt was most important, without influence from the interviewer (i.e., JV), while providing data that would address the aim of the study. Example questions and potential prompts can be seen in Table 2 below, and the full interview schedule is available in the Supplementary Materials.

### 2.3. Procedure

Advertising material was sent to the publicly available email addresses of AA training organizations. Participants were encouraged to respond either by phone or email using the contact details provided in the advertisement. All potential participants enquired via email and were sent the PICF and asked to review it. Upon returning the signed PICF, an interview was scheduled.

The interviews were conducted over Zoom (Zoom Communications, Inc., San Jose, CA, USA) by author JV and lasted approximately 40 min (M = 42.8 min, SD = 13.8 min, range = 24.31–61.42 min). Before each interview, the participant verbally assented to participate, and interview questions were then asked according to the semi-structured schedule to maintain consistency and comparability. At the conclusion of the interview, participants were reminded of mental health support links available to them if they felt that the interview had brought up any negative feelings.

The interviews were audio-recorded and then transcribed verbatim, and all identifying information (e.g., name of the participant and any colleagues, name of the organization where the participant worked) was removed to maintain privacy. Readability was enhanced by removing repeated and extra words [29]. Recordings and transcriptions were shared with the research team to cross-check transcription accuracy. Participants were supplied with a copy of their transcribed interview and were given two weeks to review the document and request any changes or clarifications. None of the participants withdrew or requested any changes, indicating that the transcripts and their responses were accurate.

### 2.4. Data Analysis

**Figure 1:**
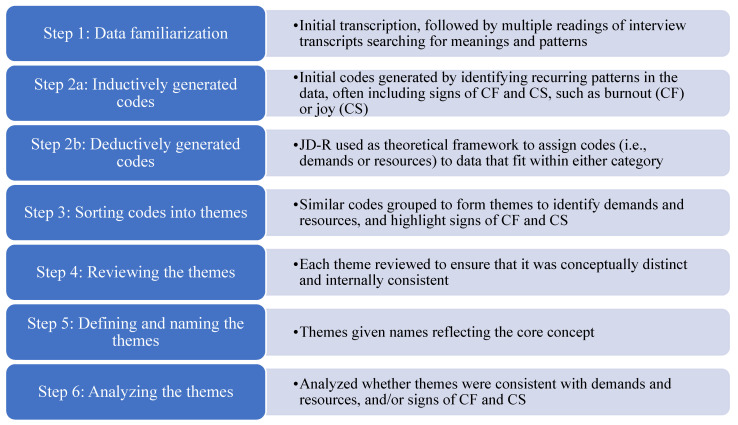
Application of Braun and Clark’s [30] six-step thematic analysis in the current study.

Data analysis followed Braun and Clarke’s [30] six-step thematic analysis. As this study employed a theoretical framework to explore demands and resources using the JD-R, yet also was exploratory in nature due to the intention to explore signs of CF and CS in the sample, both inductive data-driven analysis and deductive theory-driven analyses were performed, as discussed by Braun and Clarke [31]. This involved familiarization with the data, assigning key data pieces with a code, sorting the coded data into themes, reviewing the themes for consistency and validity, defining and naming the themes, and analyzing the themes. The steps involved in the context of this study are depicted in Figure 1.

The research team met at each step of the process to review and discuss the soundness of the analysis, especially the coding, the development of the themes, and thematic review. No decisions were made until a consensus was reached among the team. This was to ensure that the analysis was conducted in a systematic manner to produce dependable results.

## 3. Results

Five main themes were identified: Demands, Resources, Negative Impacts, Positive Impacts, and Misguided Trainer Expectations. The Demands and Resources themes were divided into several subthemes. The Demands subthemes include Emotional Demands, Cognitive Demands, and Industry Dissatisfaction. The Resources subthemes include Personal Resources and Job Resources. Quotes, corrected for grammatical errors [29] and illustrating the themes and subthemes, are presented in Table 3 below, with a brief discussion following, to provide additional detail on each theme and subtheme.

### 3.1. Theme 1: Demands

The Demands theme consisted of instances where the participants described mentally difficult or demanding aspects of their job. These fell into three subthemes: Emotional Demands, Cognitive Demands, and Industry Dissatisfaction.

#### 3.1.1. Subtheme: Emotional Demands

The Emotional Demands subtheme includes situations the trainers found emotionally taxing. Several forms of emotional demands were reported in each interview, making it the most commonly reported demand overall. Reports included concern for the wellbeing of an AA or client, exposure to client trauma, guilt when a dog did not meet training goals, and being mistreated by clients. For example, one participant described how clients could be “abusive” and stated, “I have had people shout at me. I have had people say things that are perhaps not terribly nice.” (P6). Other participants described clients trying to contact them “at 2:00 and 3:00 in the morning” (P5), “overstep [boundaries]” (P1) or “blame [them] for not fixing [the dog]” (P1). Multiple participants described instances of being exposed to their client’s trauma through “trauma-dumping” (P1).

The animal welfare concerns expressed by participants were part of this subtheme. Some of these comments were about other training providers. For instance, Participant 2 noted, “I have noticed, especially on the Facebook pages, quite a few independents who are training their dogs and the dogs are actually being abused.” Other comments were about the clients themselves, who may have had outdated beliefs about the role of an AA, and the human-animal relationship more generally, as mentioned by Participant 1:

*There are some barriers for me, I guess in the old school way of thinking of what assistance dogs do. Less [the] sort of welfare approach of creating a robot to come in and sort of do these three things or four things, and then that’s it, and the dog’s just sort of lost all of its personality. I personally really put the welfare of the dog first, so I want the dogs to be dogs first and then they help us second.*

Despite these instances, participants still expressed deep concern and worry for their clients, “You feel so sorry for them and so bad for them because of the challenges that they face” (P6). Multiple participants described “feeling really guilty” (P5 and P6) about failing or withdrawing teams that were not working out.

#### 3.1.2. Subtheme: Cognitive Demands

The Cognitive Demands subtheme includes participants’ descriptions of the cognitive exertion required for the job. Some form of cognitive demand was reported in most interviews, and usually related to high cognitive load, due to intense demands on attention, multitasking, and problem-solving. For example, “Having the mental load of just ensuring the safety of the team, […] is mentally exhausting.” (P4). Other participants describe having to be “hypervigilant” (P2) to watch out for potential dangers or feeling “drained” (P1) while problem-solving for their clients.

#### 3.1.3. Subtheme: Industry Dissatisfaction

The Industry Dissatisfaction subtheme reflected additional strain caused by dissatisfaction relating to industry matters. Most participants expressed at least one aspect of the industry that they were dissatisfied with, including unclear legislation, “[legislation requires] training to [a] standard but that standard is not defined anywhere.” (P6), and lack of regulation, “Anybody can set themselves up as a dog trainer, with no qualifications, nothing, and that’s not right.” (P6). Participants also expressed concern about the National Disability Insurance Scheme (NDIS), an Australian government program that provides individualized support plans to people with a disability [32]. According to participants, NDIS red tape was problematic, “Now there’s just red tape […] then we have to go back and explain that this person’s totally blind.” (P3). NDIS funding issues were also noted, “Thousands of dollars goes to writing up the need [by the occupational therapists and psychologists][…] That there’s no money left in their packages for anything else.” (P2). Participants expressed how these issues caused additional challenges for them and expressed a desire for more government interest in “cleaning up the industry” (P5).

### 3.2. Theme 2: Resources

The Resources theme reflected instances when the participants described something that assisted them in completing their job. These fell under two subthemes: Personal Resources and Job Resources.

#### 3.2.1. Subtheme: Personal Resources

The Personal Resources subtheme reflected participants’ descriptions of personal characteristics that assisted them in better meeting the job demands. Multiple forms of personal resources were described in each interview, including characteristics such as assertiveness, self-efficacy, and resilience. Several participants described how assertiveness assisted them in “setting boundaries” (P1), and how resilience assisted them in coping with situations others found difficult, “I think I’m kind of good at being able to separate the human side of it and detach from it quite well.” (P5). Furthermore, participants displayed self-efficacy by calming “stressed” (P1) clients with their confidence that they can achieve their goals.

#### 3.2.2. Subtheme: Job Resources

Job Resources reflected aspects of the participants’ job that assisted them. This was mostly in the form of job autonomy, which was expressed by all participants. This could be observed when participants described being able to set their own schedules, take their child to work (P5), or just stop to cuddle dogs for a while. Additionally, two participants reported job resources such as supervisor feedback and workplace training. One participant also described organizational support including “two psychologists on staff” (P3), and supervisors and coworkers who are “very trusting and supportive” (P3). In contrast, one participant described a lack of job resources, “I’ve even driven away from a session and gone, “God, I don’t know what to do there.” […] “I wish I had someone to bounce ideas off of.”” (P1). This participant went on to explain that they were able to deal with the demands but had to rely on personal resources instead.

### 3.3. Theme 3: Negative Impacts

The Negative Impacts theme reflected participants’ descriptions of how they were impacted by the demanding aspects of their jobs. Most participants reported experiencing some negative impact, many of which matched signs of CF, including stress, burnout, feeling overwhelmed, exhaustion, mental health struggles, and ruminating on their clients. Two participants reported experiencing “burnout” (P1 and P3). One of these participants (P3), as well as another participant (P4), reported experiencing small amounts of CF, “I think compassion fatigue definitely kicks in with the clients, but it definitely kicks in with the dogs as well.” (P3). These reports of CF were self-assessed, and were brought up unprompted, before the interviewer defined and specifically asked about CF.

All participants agreed that CF was an issue among trainers, after being given a brief definition of CF from the interviewer and asked for their thoughts. One participant then said that a specialist had suggested that they may have CF during a previous job, how damaging it was, and how they needed to be careful not to end up “back there again” (P2).

### 3.4. Theme 4: Positive Impacts

The Positive Impacts theme reflected participants’ descriptions of how they were impacted by the rewarding aspects of their jobs. Many of these impacts matched signs of CS, with participants unanimously reporting feelings of fulfillment, joy, satisfaction, love for their job, and motivation from helping people. Every participant expressed fulfillment by describing how they felt when they helped their clients to grow and achieve things that they didn’t think they could, this was described as, “beautiful” (P1), “amazing” (P5), “very rewarding” (P6). Participants also unanimously expressed “loving” the job. One participant also described “maturing” in the job and stated, “It definitely changed the way I see things and who I am as well.” (P4).

Participants described the satisfaction of helping people as their main motivation. This can be seen in quotes such as, “That’s what keeps us in [the job]” (P6), “That is what drives me to keep going.” (P2), and “They are my reason for continuing on.” (P1). Moreover, all participants agreed that they experienced CS regularly after being given a brief description of CS.

### 3.5. Theme 5: Misguided Trainer Expectations

The Misguided Trainer Expectations theme reflected the expectations trainers had starting the job compared to the reality of the role. Most participants started as animal trainers without a background in human services. Some were expecting to work mainly with animals, “I probably was naive in the beginning and thought it was very much just, go in and train the dog” (P1), but found that “I more work with the humans over the dogs.” (P1). They continued, “I’ve had three different trainers come through and go, because the human end was too much, they didn’t expect it to be as big as what it was.” (P1). This participant felt that there was a “misunderstanding” regarding the role and how “human focused” the role is.

## 4. Discussion

This study aimed to qualitatively identify the job demands and resources of AA trainers and explore whether signs of compassion fatigue (CF) and compassion satisfaction (CS) exist in these workers. Through interviews with six participants, we identified five themes: Demands, Resources, Negative Impacts, Positive Impacts, and Misguided Trainer Expectations. The Demands and Resources themes included a range of job demands and resources experienced by AA trainers, while the Negative and Positive Impacts themes provided evidence for signs of CF and CS, respectively.

### 4.1. Demands and Resources Themes

The Demands theme included experiences that participants reported as challenging or demanding, while the Resources theme included personal factors or job aspects that assisted them to better meet or cope with the job demands. Job demands fell into three subthemes: Emotional Demands, Cognitive Demands, and Industry Dissatisfaction. The Emotional Demands subtheme included situations the trainers found emotionally taxing, including concern for the wellbeing of an AA or client, exposure to client trauma, guilt when a dog did not meet training goals, and being mistreated by clients. These emotional demands can have negative impacts on workers; for example, exposure to client trauma or suffering is a significant predictor of CF (STS and burnout) among mental health professionals [9]. Additionally, work-induced guilt among carers has been linked with emotional over-involvement [33] and can lead to reduced job and life satisfaction [34], while working in an abusive environment has been associated with lower job satisfaction and intentions to leave [35].

Emotional demands were the most commonly reported demands by AA trainers, with some associated negative outcomes, such as burnout, also reported by trainers. This indicates that emotional demands may be a particularly important factor to consider regarding the potential development of CF among AA trainers, but further research is required before any conclusions can be made.

The Cognitive Demands subtheme included participants’ descriptions of the cognitive exertion required for the job, which was usually related to high cognitive load. High cognitive load has been associated with burnout [36] and exhaustion [37]. Both burnout and exhaustion were reported by participants, with one participant attributing their exhaustion directly to mental load. This suggests that cognitive demands may also be an important factor regarding the potential development of CF among AA trainers.

The Industry Dissatisfaction subtheme reflected additional strain caused by dissatisfaction relating to industry matters. This included lack of regulation, unclear legislation, NDIS (i.e., an Australian government agency providing individualized plans to support people with a disability) red tape, and NDIS funding issues. Some of these issues have been directly investigated, such as additional red tape being associated with lower job satisfaction [38], while other issues have not been explored. However, it is possible that funding issues may represent job insecurity for trainers, which has been negatively related to wellbeing [39]. Also, unclear legislation may represent role ambiguity, which has been associated with poorer performance [40]. Similar themes such as lack of regulation, unclear legislation, and funding barriers were also noted as some of the main service delivery barriers for AA trainers by Hill et al. [23]. The authors highlighted the need for clearer legislation, especially regarding training requirements for AA providers.

Participants reported a range of experiences that were identified as job Resources, these fell under two subthemes: Personal Resources and Job Resources. The Personal Resources subtheme reflected participants’ personal characteristics that assisted them in better meeting the job demands, such as assertiveness, self-efficacy, and resilience. These resources can have positive impacts on trainers; self-efficacy has been found to reduce burnout and increase CS [41], while resilience has been negatively associated with exhaustion [42]. Personal resources were the most commonly reported resources by AA trainers. This indicates that personal resources may also be a particularly important factor regarding the potential development of both CF and CS among AA trainers.

The Job Resources subtheme reflected elements of the participants’ working roles that assisted them, including job autonomy, supervisor support, and workplace training. Job autonomy has been associated with improved job performance and satisfaction [43] and was reported by every trainer. While organizational supports such as supervisor support and training have been identified as the most commonly reported job resources in reducing CF among mental health professionals [9], they were only reported by two AA trainers, with one trainer even reporting a lack of support. This indicates that, while job autonomy may be an important factor regarding the potential development of both CF and CS among AA trainers, other job resources such as supervisor support and training may be lacking. More research is needed to better understand the organization-based support offered to AA trainers.

### 4.2. Negative and Positive Impacts Themes

The Negative Impacts theme reflected how participants were negatively affected by their demanding experiences, and many of these impacts matched signs of CF. The Positive Impacts theme reflected how participants were positively affected by aspects of their work, and many of these impacts matched signs of CS.

The reported negative impacts included stress, burnout, feeling overwhelmed, exhaustion, mental health struggles, CF, and ruminating on their clients. Stress, exhaustion, and feeling overwhelmed are all signs of burnout [8] and were reported by multiple participants. Furthermore, two participants of this study reported experiencing burnout, per se. Demands that were identified in both the Emotional and Cognitive subthemes have been associated with the development of burnout and may indicate potential factors related to the development of burnout among AA trainers. This includes high mental load [36] and exposure to the trauma of a client, which has been identified as a predictor of burnout [9]. Burnout has been associated with an increase in staff turnover [44], and a decrease in quality of life among nurses [45]. As burnout is a component of CF [8], the identification of burnout among participants provides some evidence of CF among AA trainers.

While no participants directly reported experiencing secondary traumatic stress (STS), the other component of CF, multiple participants reported ruminating on their clients, which could indicate a sign of STS. Exposure to the trauma of a client, one of the emotional demands identified in this study, has also been found to predict STS [9], which may indicate a potential for STS to exist among AA trainers, but the evidence for this is limited.

Two participants directly reported experiencing small amounts of CF even before being provided with the definition later in the interview, but these were self-assessed reports only and did not include the use of an assessment tool or a formal diagnosis. Therefore, while this is an additional sign of CF among AA trainers, without research involving the use of a valid and reliable screening tool, the conclusions that can be drawn from these reports are limited.

The reported positive impacts included feelings of fulfillment, joy, CS, love for the job, and motivation from helping people. These impacts were unanimously reported by all the participants. Psychological boosts such as fulfillment and joy derived from helping people can lead to CS [46]. CS is associated with increased employee retention and job satisfaction [47]. All participants agreed that they regularly experienced CS from their job, after being given a brief description of CS. This provides evidence of the existence of CS among AA trainers.

While this study identifies CS as a positive impact of the job, CS is also considered a personal resource that has been found to have a buffering effect against high job demands [48]. Moreover, CS can act as a buffering factor against CF [49]. This can reduce the impact of the negatives associated with helping and lower the risk of CF [9]. Therefore, CS could also be considered a personal resource and an important factor regarding the potential development of CF among AA trainers.

This study identified signs of both CF and CS among trainers. When considered through the framework of the JD-R (i.e., job strain occurs when demands are too high and resources are insufficient [26]), this may indicate that job demands are high, and strain is occurring. Furthermore, while there are sufficient resources to develop motivation and potentially CS, they may not always be sufficient to buffer the impacts of the demands. Moreover, multiple personal resources were commonly reported by each participant, while job resources were less commonly reported. This may mean that additional job resources are required to reduce job strain and improve AA trainer wellbeing. Future research should investigate this possibility.

A similar combination of high stress and high satisfaction has been observed in direct-care disability support workers [50]. Animal healthcare workers also report high levels of CF and CS [51]. However, workers who were in a position of high stress and high satisfaction were identified as potentially vulnerable as they were likely to persevere in their role despite high stress levels [50]. It is possible that AA trainers are experiencing something similar, and that care should be taken to reduce the risk of CF even if CS is present.

### 4.3. Misguided Trainer Expectations Theme

The final theme was an unanticipated finding that emerged during inductive analysis and did not directly relate to the JD-R framework, CF, or CS, but still has important implications for the field. The Misguided Trainer Expectations theme reflected the expectations trainers had at the beginning of their career that the role would mostly involve working with dogs; however, it actually involves mostly working with people. Unmet job expectations can lead to emotional exhaustion and higher staff turnover [52]. This has implications for training organization recruitment, and how they set expectations for new trainers before hiring. It is important for organizations to clearly explain, early on, that the role of an AA trainer has a strong human services component and is not exclusively about training the dogs.

### 4.4. Implications of Findings

The identification of AA trainer demands, resources, and signs of CF and CS has practical applications for training organizations. Organizations can use these findings to better support trainers by offering programs such as wellbeing education and training, and mentorship. Promoting practices that increase CS to improve employee wellbeing, along with education regarding the risks of CF, including how to monitor for symptoms and how to set up self-care routines, are the first steps in preventing CF [53]. Additionally, supervisor or mentor support can improve quality-of-life measures [9], and programs such as mental health first aid training in other helping professionals can improve personal mental health outcomes among workers, and better outcomes for clients [54].

The identification of the job demands and resources in AA trainers has theoretical applications for future research. These findings provide the foundation for building a JD-R model specific to AA trainers. A job-specific JD-R model could be used, for example, to predict CF based on which demands trainers are exposed to, or to identify which resources provide the most support to trainers.

Finally, the discovery of signs of CF provides evidence of a previously unidentified psychosocial hazard for trainers. CF has a risk of increasing worker attrition, which in this case could lead to fewer AAs being trained and longer wait lists for people in need. CF would likely be considered a psychological risk, meaning that employers of AA trainers would be required to provide protections to reduce the likelihood of CF.

### 4.5. Limitations and Future Research

Some limitations of this study should be acknowledged. All participants were currently working as AA trainers. This study was unable to recruit any participants who had recently left the profession. These trainers may have provided valuable insights into how their job had impacted them and what caused them to leave. Also, the sample was small, but we believe that data saturation was reached due to a lack of new information emerging from the later interviews.

An additional limitation was that only assistance dog trainers participated in this study. AAs come in many different species (e.g., cats, miniature horses), and it is possible that other trainers may have completely different experiences. This limits the generalizability of these findings to assistance dog trainers, rather than AA trainers more broadly, although dogs are the most common species employed for this type of work, so most AA trainers likely work exclusively with dogs. Furthermore, we did not ask participants to describe their professional background or relevant education. This is because Australia has no formal requirements for becoming an assistance dog trainer. Therefore, it is possible that there is a range of different educational and professional backgrounds among our participants which was not captured in the current data.

While this study identified signs of CF and CS among the participants, this is not a confirmation of the existence of the phenomenon. Future quantitative research using a CF and CS screening tool, such as the Professional Quality of Life Scale (ProQOL) [8], is warranted as a next step. This is a self-report measure that would be useful in larger-scale research in this population, but it was not deemed appropriate for the current study due to its exploratory nature. Additionally, researchers could operationalize and incorporate the identified demands and resources into scales and questionnaires, which could then be used to identify any specific demands or resources predicting either CF or CS, along with which interventions offer the most protection.

## 5. Conclusions

This qualitative interview study investigating AA trainer job demands and resources was based on the rationale that AA trainers’ job experiences were not well understood, and that, as helping professionals who are potentially being exposed to trauma and suffering, they may be experiencing CF. This study identified a range of demands and resources and identified signs of both CF and CS. These findings have practical applications for training organizations looking to support their trainers, and the identification of signs of a previously unidentified psychosocial hazard in this profession. The negative outcomes associated with CF could result in a lower quality of life for trainers, fewer trainers, and longer wait times for people in need. CF has negative outcomes for those who develop it, and this research may lead to greater awareness and future workplace interventions to assist AA trainers.

## Figures and Tables

**Table 1:** Information about the participants and their working roles.

Participant Number	Gender	Employment Status
P1	Female	Small training organization owner—full-time
P2	Female	Sole trader—full-time
P3	Male	Large training organization employee—full-time
P4	Male	Large training organization employee—full-time
P5	Female	Sole trader—full-time
P6	Female	Small training organization employee—part-time

**Table 2:** Example interview questions and prompts.

Question type	Example Questions	Example Prompts
Exploration	Could you tell me about the populations that you generally work with?	What are some of the presenting issues of the handlers you work with?
Demands exploration	What are the demanding or stressful aspects of your job?	Can you tell me why these aspects are demanding or stressful?
Impacts exploration	What impact do these aspects have on you?	Impact on your wellbeing, how you feel, act, or behave in or outside of work
Compassion fatigue and satisfaction definitions	There is a phenomenon called compassion fatigue that is sometimes experienced by people who work in helping professions. It can include exhaustion and preoccupation with the suffering of others, as well as symptoms such as feeling overwhelmed, sleep disturbances, nightmares, and a quick temper. Is this something that you can relate to or believe may be an issue among animal assistance trainers?	Can you provide some examples?

**Table 3:** Participant quotes illustrating the core content of each theme and subtheme.

Theme	Subtheme	Illustrative Quote
Demands	Emotional demand—guilt	Oh, withdrawing teams if they’re not suitable, I find that really difficult to do. Yeah, I feel really guilty. (P5)
	Emotional demand— animal welfare concern	“…having to implement boundaries of the welfare of the dog over and over and over and over again…” (P1)
		The dog jumped up on me to say hello and I said, “Hello sweetie pie.” I’ll get her into a sit. Before he can have a chance to get the poor dog into a sit, he just grabs the dog, picks it up, slams it down on the ground. Lays on top of it and growls in the dog’s face. And I’m thinking, “What the hell.” (P2)
	Emotional demand—trauma dumping	There can be a lot of trauma-dumping [from clients] […]. All of a sudden, you’re walking out going, “I didn’t need to know all of that information, that was not necessary for me.” and it can stick with you. (P1)
	Cognitive demand— hypervigilance	Having to be hypervigilant watching what’s going on, watching the dog, watching the person, making sure that everybody is gelling together. (P2)
	Cognitive demand— cognitive load	Dealing with stresses while you’re not feeling 100% yourself is definitely something that comes into it, and just the mental load as well. (P4)
	Industry dissatisfaction—Lack of regulation	I really get frustrated about, there are certain organizations where a majority of those dogs are really not very well trained at all. (P2)
	Industry dissatisfaction—Unclear legislation	There’s also a bit of frustration at the messiness of the industry and the inconsistencies, so it can be quite demanding. (P5)
Resources	Personal resources— assertiveness	Boundaries is what helped the challenging moments which were really people trying to overstep. (P1)
	Personal resources— self-efficacy	They might have been stressing about this one thing that their dog has done […]. Then you come in and you just go, “It’s OK, everything’s fine […] we’ve got a path through.” (P1)
	Personal resources— resilience	Some people can be difficult to deal with, that’s where the nursing background comes in, I can utilize a lot of that. (P6)
	Job resources—co-worker support	We have very open-door policies for all our managers and in terms of communicating, […] we’re a very trusting team. (P3)
	Job resources— job training	[Work] put us in a mental health first aid course […] it definitely gives you the ability to have a bit more of awareness of what the signs are […] and how to offer them support. (P3)
Negative impacts	Compassion fatigue (incl. burnout)	They all have their own stories and some of them are quite difficult, so dealing with the knowledge of that and the implications, as well. So, taking on the handler’s stories and that compassion approach, you can get that compassion fatigue as well. (P4)
		It [CF] is definitely out there, and I do see it with others, and people certainly leave the industry because of it. (P6)
		I was just done by the end of it, I had nothing left in the tank for me. […] I didn’t focus on my mental health, […] that’s what led to my burnout moments. Because there are some people that you work with that are super easy and then there are others that are just sticky and it’s like you come home and you hold it, you feel it for the rest of the day. (P1)
		So, I still struggle with burnout every now and then. But yeah, in the beginning it was a lot worse. I would get very overwhelmed. (P1)
Positive impacts	Happiness	I am so much happier. I’m sleeping well, you know, I’m happy, I go out socializing now with my friends, we go out for lunches. (P2)
	Fulfilling	[child client] wouldn’t talk, he wouldn’t walk anywhere, he would do nothing […he became] a child that was confident, it was just amazing and building that up with that child was absolutely incredible. (P2)
	Satisfaction	That satisfaction is definitely there, and it shows up daily almost now, with most clients. (P1)
Misguided trainer expectations	Working with people more demanding than expected	It can be a bit more demanding than I thought it would have been. I’m pretty okay as far as the dog training side of it is concerned, but then some people can be difficult to deal with. (P6)

## Data Availability

Data are unavailable to share due to privacy and ethical constraints.

## Supplementary Materials

The following supporting information can be downloaded at: https://www.mdpi.com/article/10.3390/ani15030337/s1, Interview Schedule.
