# Supplementary material for: Factors Associated with Compassion Fatigue in Assistance Animal Trainers in Australia—A Qualitative Investigation
Source: Animals (Basel). 2025 Jan 24;15(3):337. doi: 10.3390/ani15030337 (PMC11816333; doi:10.3390/ani15030337)
Supplement: Supplementary file 1 [file animals-15-00337-s001.zip › animals-3377896-supplementary.pdf]

Interview Guide

1. Can you tell me about why you became an AA trainer?
2. Can you give me an overview of what being an AA trainer involves?
3. How do you feel about your job?

   (PROMPTS: Does it match your expectations? – Why, why not?)
4. Could you tell me about the populations that you generally work with?

   (PROMPTS: Children, teens, adults?)

   What are some of the presenting issues of the handlers you work with?
5. What are the demanding or stressful aspects of your job?

   (PROMPT) interactions with animals, handlers, colleagues, policies

   (PROMPT) Can you tell me why these aspects are demanding or stressful?

   (PROMPT) Is there anything else?
6. What impact do these aspects have on you?

   (PROMPT) impact on your wellbeing, how you feel, act, or behave in or outside of work
7. What's rewarding about your job?

   (PROMPT) interactions with animals, handlers, colleagues, policies

   (PROMPT) Can you tell me why these aspects are rewarding?

   (PROMPT) Is there anything else?
8. What impact does that have on you?

   (PROMPT) impact on your wellbeing, how you feel, act, or behave in or outside of work
9. Are there any barriers that makes it harder for you to complete your job?
   - Could you tell me about some of them?
10. What would provide the biggest assistance to you in reducing stress and improving wellbeing?
    - Is there anything you can think of that the organisation could do to support you?
11. There is a phenomenon called compassion fatigue that is sometimes experienced by people who work in helping professions. It can include exhaustion and preoccupation with the suffering of others, as well as symptoms such as feeling overwhelmed, sleep disturbances, nightmares, and a quick temper. Is this something that you can relate to or believe may be an issue among animal assistance trainers?
    - (If yes) Can you provide some examples?

12. There is another phenomenon called compassion satisfaction that is sometimes experienced by people who work in helping professions. This is the positive feelings associated with helping people, like the pleasure or fulfillment that individuals derive from doing their work. Feeling positively about colleagues, helping someone in need, or helping the organisation or wider community, can all lead to compassion satisfaction. Is this something that you can relate to or believe may be common among animal assistance trainers?
    - (If yes) Can you provide some examples?

Thanks again for allowing me to interview you today. Just as a quick reminder, if this conversation brought up any negative feelings, you can contact Beyond Blue or Lifeline. I'm putting those numbers and websites into the chat function now.

Beyond Blue: https://www.beyondblue.org.au/ or 1300 22 4636

Lifeline: https://www.lifeline.org.au/ or 13 11 14
